# Synergetic synchronized oscillation by distributed neural integrators to induce dynamic equilibrium in energy dissipation systems

**DOI:** 10.1038/s41598-022-21261-w

**Published:** 2022-10-13

**Authors:** Mitsuhiro Hayashibe, Shingo Shimoda

**Affiliations:** 1 Department of Robotics, Graduate School of Engineering, Tohoku University, Sendai, Japan; 2 RIKEN Center for Brain Science, TOYOTA Collaboration Center, RIKEN, Nagoya, Japan

**Keywords:** Statistical physics, thermodynamics and nonlinear dynamics, Nonlinear phenomena

## Abstract

The synchronization phenomenon is common to many natural mechanical systems. Joint friction and damping in humans and animals are associated with energy dissipation. A coupled oscillator model is conventionally used to manage multiple joint torque generations to form a limit cycle in an energy dissipation system. The coupling term design and the frequency and phase settings become issues when selecting the oscillator model. The relative coupling relationship between oscillators needs to be predefined for unknown dynamics systems, which is quite challenging problem. We present a simple distributed neural integrators method to induce the limit cycle in unknown energy dissipation systems without using a coupled oscillator. The results demonstrate that synergetic synchronized oscillation could be produced that adapts to different physical environments. Finding the balanced energy injection by neural inputs to form dynamic equilibrium is not a trivial problem, when the dynamics information is not priorly known. The proposed method realized self-organized pattern generation to induce the dynamic equilibrium for different mechanical systems. The oscillation was managed without using the explicit phase or frequency knowledge. However, phase, frequency, and amplitude modulation emerged to form an efficient synchronized limit cycle. This type of distributed neural integrator can be used as a source for regulating multi-joint coordination to induce synergetic oscillations in natural mechanical systems.

## Introduction

Human and animal motions are typically composed of rhythmic components such as the flapping of bird wings and the locomotion of vertebrates. Because the spinal nervous network generates and synchronizes these rhythmic components, humans and animals can walk and run, fish can swim, and insects can crawl by interacting with the environment^1^. Because the synchronization phenomenon is common to many natural mechanical systems, the synchronization between different periodic systems is an attractive scientific topic. A famous example is Huygens’ synchronization observations for two pendulum clocks^2,3^. This finding has served to inspire studies on sympathetic rhythms of interacting nonlinear oscillators in many areas of science. The particular phase relations in the synchronization process are a fundamental problem of nonlinear dynamics^4–6^. Studies that have been fervently pursued in recent years range from neurobiology and brain function to human behaviour^7–10^. In Huygens’ clock, a pendulum is attached to an escapement mechanism, which alternately blocks and releases a scape wheel as the pendulum oscillates. This action provides the timing that is transmitted from the scape wheel to the clocks’ hands via a gearing system. The scape wheel is also connected through a separate gearing system to an energy source, typically a wound spring or elevated weights. The energy system drives the motion of the scape wheel, which in turn provides small “kicks” to the pendulum via the escapement. This transmission of energy to the pendulum compensates for losses due to friction; thus, the pendulum continues to oscillate indefinitely as long as the spring is periodically rewound or the fallen weights are periodically raised back up^2^. Huygens’ clock synchronization and its limit cycle can be thus be generated purely by passive mechanical interactions between two clocks. The escapement mechanism compensates the lost energy to dissipate non-energy to enable the pendulum system to have the same amplitude oscillation. In contrast, real-world mechanical joints in humans and animals are associated with energy dissipation by joint friction and damping. The damped joint is a source of energy loss in natural systems. In order to sustain rhythm without attenuation, it is necessary to constantly supply energy to the articulated joints by actively applying torque through joint actuation itself. Because the lost energy at the joint and the actively injected energy with the joint torque should be well equilibrated, the management of multiple joint torque generations to form a limit cycle in an energy dissipation system is a difficult problem. If the injected energy is less than the dissipated energy, the system will be stopped at one time. If the injected energy is greater than the lost energy, it can be diverged. Although this issue can be managed in animal systems to form limit-cycle oscillations, it is computationally difficult in an unknown environment when no explicit dynamic information is given a priori. Recently considered to be embedded in our spinal system, central pattern generators (CPGs) are dynamical systems exhibiting limit-cycle behaviors^11–13^. The task of creating the basic pattern of repetitive movement is delegated to the nervous system at the spinal level in the central nervous system. The current state-of-the-art approach to computationally represent the functionality of CPGs involves the use of a coupled oscillator model^4,14,15^. Coupled oscillator models have been used for different motion generation purposes^16–18^. A coupled oscillator model takes the form of nonlinear derivative equations along with the coupled relationship to the neighboring oscillator in general^19^. This means that the relative dynamic structure between neurons is embedded manually by the selection of an oscillator model among different types of nonlinear models. The tuning parameters, such as frequency, amplitude, and phase, can still be flexible. However, the inhibitory, excitatory, or phasic relationship which forms the coupling, is mathematically embedded in the relative relationship between neurons from the beginning. For instance, in the case where the Hopf oscillator model^15,20^ is employed, knowing if the dynamics of this Hopf oscillator are reasonable for the coordination of a specific system can be difficult to determine. When the dynamics of the body environment are essentially unknown, it is difficult to select a nonlinear model. The success of forming the limit-cycle oscillation with coupled oscillators lies in their capability to control the interaction forces between the joints or neurons by using phase information or by the coupled term for regulation of the system. When the distance of the phases increases for the predefined coupled term in the oscillator, such a coupled term can increase the torque to avoid having more phase gaps of the joint motions, and vice versa. This means that energy injection or reduction to induce synchronization is manually embedded in coupled oscillators using the phase difference information or using the embedded coupling terms. This coupled term drives the control of the energy injection balance even when energy is lost due to damping of the joint or by the effect of friction with the environment. However, the system behavior is highly dependent on the design of the coupled structure in the oscillator model. If the system dynamic information is not given a priori, the coupled oscillator cannot be used.

This study aimed to verify if simple distributed neural integrators can manage self-organized pattern generation as a new method without using so-called coupled term between neighboring joint or neuron. If it is possible to induce resultant adaptive CPG-like limit-cycle patterns without a prior oscillatory assumption, it should be convincing to reveal the authentic joint coordination mechanism regarding motor control for redundant systems with damping in the joints. Here, we developed a method for synchronization in an energy dissipation system and observed the phenomenon of synchronization by a simple neural integrators approach. Energy injection was managed internally by a synergetic learning control as the decentralized control architecture. Only neural integration process is assumed without assuming prior coupling dynamics. Synchronization in the energy dissipation system was managed with the proposed decentralized control method. Here, we report on our method, and observations along with an analysis of different systems and a quantitative evaluation. We believe it is important to report this self-organized synchronization phenomenon without waiting for the mathematical proof for the sake of science.

Our learning control is designed based on four main requirements. Requirement 1: The given dynamic environment is unknown to the controller and possibly time-variant, thus the model-free approach should be taken. The model optimization approach faces the problem of an unknown time-variant environment. Requirement 2: The adaptive control should be executed in real time, with simultaneous computations for learning and control. The computation should be in a decentralized manner with a simple modular structure without using information from neighboring joint or neuron. Requirement 3: Typical oscillator models should not be used as they are influenced by the coupled dynamics of the chosen model structure. Requirement 4: The simple control should be applicable to different complex mechanics of multiple joints, such as concatenated segmental mechanics.

## Results

### Forming limit cycle

**Figure 1:**
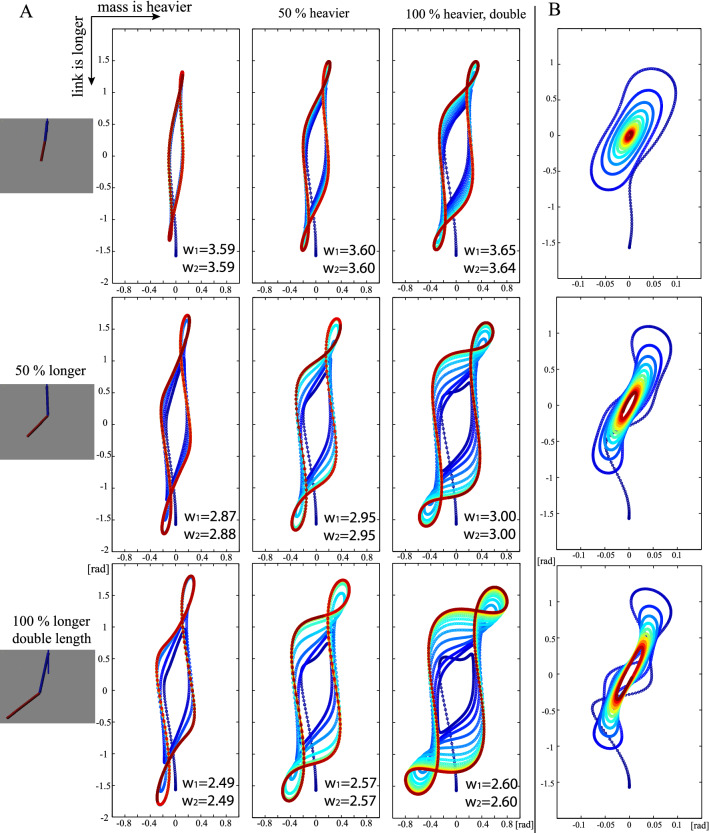
(**A**) Phase portrait of a double pendulum controlled with the proposed neural integrator in different dynamic conditions. The pendulum length and mass were changed. The dynamics setting was set blind for the controller. The first joint angle in the x-direction and the second joint angle in the y-direction. Even under different physical conditions, limit-cycle oscillations could be created, without the so-called coupled oscillator model. The converged oscillation frequency was different for different conditions. The long limb case resulted in a slow oscillation and the short limb resulted in a fast oscillation. (**B**) shows the results when the integration term was omitted for the original mass condition. From top to bottom, the length of the limb increases, which is similar to the condition on the left-hand side. Without the integration term, the energy was dissipated by damping and the oscillation could not be sustained. We find a “frequency modulation” function that corresponds to different dynamic conditions. Movie S1 shows the simulation results for this result.

The connected segment dynamics is known as a highly nonlinear and chaotic system. A double pendulum is an example that exhibits chaotic behavior with a strong sensitivity to initial conditions^21^. Maintaining oscillations in such systems requires synergetic articulation of the joints. In particular, when the joint exhibits a damping effect, the amplitude of the oscillation is reduced and the lost energy should be compensated with the active torque of the joint. When the dynamics equation is available, it is possible to solve with an analytical approach with mathematical optimization. In another manner, a coupled oscillator model that generates interaction torque using phase difference or using coupled dynamics can specify the force interactions over joints^22–24^. However, when such dynamics equation is not available, it is not possible to solve the problem with existing approaches. We begin our study by considering a double pendulum in which both joints had a certain level of damping and the second joint is only actuated. Thus, the problem is how to produce the limit cycle for both joints with only one controllable joint. As the system has complex interaction torque dynamics between the joints, the active joint should be coordinated to achieve synergetic synchronization for the total system^25^. Figure 1 shows the result of double pendulum control in different physical conditions. The pendulum length and mass conditions are largely changed. Even with different initial conditions, the same limit-cycle behavior was produced with the same frequency over the two joints. The neural integrator was added only to the 2nd joint, the second link was actively controlled, and the first link was completely passive. Although we could not directly specify the motion frequency for the first link, the oscillation frequency of the 1st joint and 2nd joint was matched. We refer to this as synchronized oscillation. Similar to swing seat oscillation, the movement of the human knee coordinates the lower limb synergistically to the whole swing seat oscillation, thus resulting in the same oscillation frequency with a certain phase delay. This result indicated that limit-cycle oscillation can be created by neural integration without using the so-called coupled oscillator model. The converged limit-cycle oscillation speed varied by physical conditions. In our approach, because there is no parameter to specify the frequency, it is found in a natural manner. In the controller, because there is no frequency parameter, a Fourier analysis is used on the resulting joint angle to evaluate the angular velocity. The long limb case was slow and the short limb was fast. The frequency information is shown for each link is shown in Fig. 1, demonstrating the same value over links. The obtained frequency approximately alters with scaling with the square root of the limb length and did not change for the mass condition. The energetically reasonable frequency for forming the limit cycle of the given system was found following natural mechanical system. It is necessary to find the reasonable “kick” to the system for different pendulum lengths and inertia situations as in swing seat oscillation. Finding a reasonable way to control the “kick” in an unknown dynamics environment is difficult. The right column of Fig. 1 shows the result when the learning term forming the feedforward command was switched off. We can observe that the phase area is shrunk and will stop by losing energy if there is only feedback control. This indicates that the added neural integration term allows the limit cycle to be formed without having prior dynamics information.

In our article, the method was tried with many types of dynamical environment with simulation. For simulating joint dynamics, we used the Open Dynamics Engine^26^ which is popular physics engine for a rigid body dynamics simulation. All the dynamics simulations were managed by external ODE package, in which the control module has access only to the control of each joint torque, and none to the manipulator dynamics model itself.

#### Experiment on real system

To verify the control performance, the usage of physics engine is already well accepted in the research society. However, to show the proposed method is feasible for real system, the limit-cycle oscillation emergence of one pendulum was tested on the robot system. Experimental system is detailed at supplementary material. Movie S4 shows this experimental result well. The first sequence shows without neural integration only with feedback term, which can not sustain the oscillation by the damping effect. The second sequence with neural integration found the limit-cycle, and some disturbances by hand was applied to the system, but after some time, it recovers the same limit cycle. Figure S4 is corresponding to this video segment, we have verified the angular velocity and confirmed it converges to the same limit-cycle with same frequency. Even if we provide the complex interaction by the hand to the real system, it recovered into the same rhythm. This result shows well the feasibility of the method to the real system.

### Forming limit cycle over 2 pendulums

**Figure 2:**
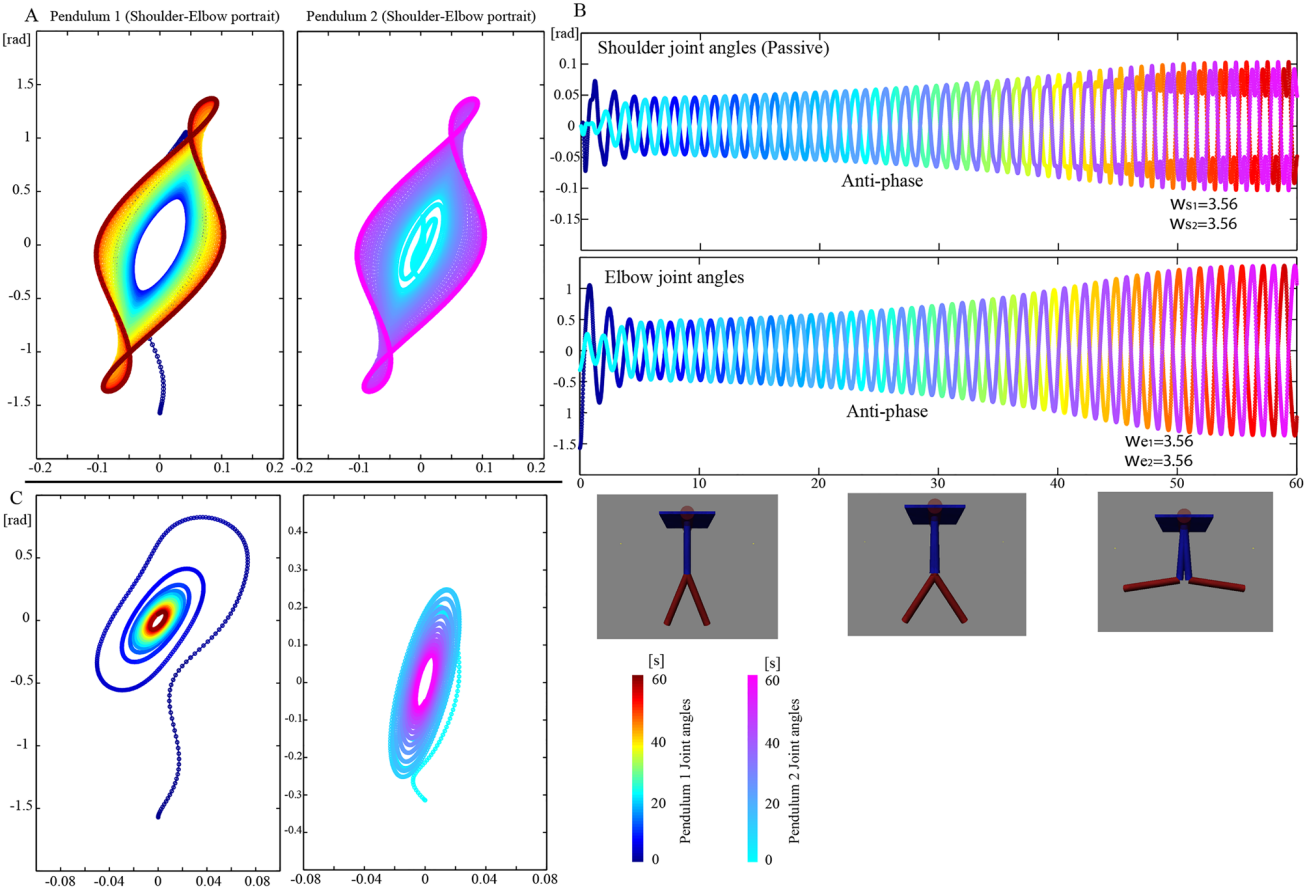
(**A**) Phase portrait of two double pendulums connected to a common floating base controlled with the proposed neural integrator. (**B**) The shoulder joint (first joint) angle and the elbow (second joint) change over time in anti-phase. The initial condition was neither anti-phase nor in-phase, and there was some phase delay between them. When the common base was fixed, there was no interaction between the rhythm of the two pendulums, and synergetic oscillation inside the pendulum as in Fig. 1 occurred independently with each pendulum following its own rhythm. When the common base was floating, the pendulums achieved anti-phase oscillation instantly; in this anti-phase of the two pendulums, the force interaction by the root link to the base was in the opposite direction. The base was therefore not moving, presenting a type of energy-efficient synergetic oscillation as total system. This implies that “phase modulation” could be achieved with this method, and an efficient way to sustain oscillations was found by neural integration. (**C**) When neural integration was not used, the oscillation could not be sustained.

In the next example, the given dynamics is more complex, although the double pendulum itself is already known to exhibit chaotic behavior. The two double pendulums were attached to a floating base as in Fig. 2. Because the base plate was not fixed, it could be moved horizontally if the force from the pendulum was applied. Therefore, the blue parts could be passively moved. The 2nd red link of the two double pendulums could only be actively controlled. In one double pendulum, there were only interaction forces between the two links. With the additional interaction of the two sets of double pendulums, it is difficult to decide how to control the active joints to keep the energy of the given system. With joint damping, the energy can be absorbed to move the base. Although it is necessary to discover how to move the joints without moving the base for energy efficiency, it is difficult to determine without having dynamics model information of the environment. Starting with different initial conditions, our results showed that the phase became anti-phase between the two double pendulums. The shoulder joint (1st joint) and elbow angle moved in the opposite direction between the pendulums. Inside one pendulum, the shoulder and elbow of the same pendulum was in-phase. As in Fig. 2, the initial period was nearly in-phase, but it changed to anti-phase after 5 s. Importantly, because the controller lacks phase parameters, the motion was altered purely by the proposed method. The four joint angle values and angular velocities were evaluated, and they converged to the same angular velocity between the two sets of pendulums as in Fig. 2. As the controller was a purely decentralized system, one pendulum did not know the other pendulum’s state. The controller does not know if the base is moving or not and only uses the weak feedback of the self-joint angle and the torque integration. The torque integration term acquires the energy usage of the cyclic motion. The energy-feedback effect to the total system results in an energy-preserving motor command in the joint damped system and the moving base environment. As it converges into an anti-phase one between the pendulums, the interaction torque between the base and the pendulum cancels each other and the base motion is minimized. This environmental adaptation was achieved without using a dynamics model or an oscillator model. The simple neural integrator forms a feedforward motor command to form the limit cycle in this complex dynamic environment. The left bottom graph in Fig. 2 shows the phase portrait when the integrative term is switched off. The phase area can be observed to shrink before going to stop, where it then loses energy if it is only with low gain feedback control. It is also confirmed that the form of phase portrait is independent, in contrast to the phase portrait form that is exactly the same in the case of the integration case, converges to the same trajectory of the shoulder and elbow, and is in anti-phase. The four oscillations as in Fig. 2 have the same angular frequency over four joints. This observation implies that the added term did the work to achieve synergetic synchronization in this energy dissipation system. Just by a simple neural integrator without neither frequency or phase information nor a coupled oscillator, anti-phase motion that results in limit-cycle oscillation in an energy dissipation system is an important find. Movie S2 shows the simulation result in control.

### Forming limit cycle over multi-segment system

**Figure 3:**
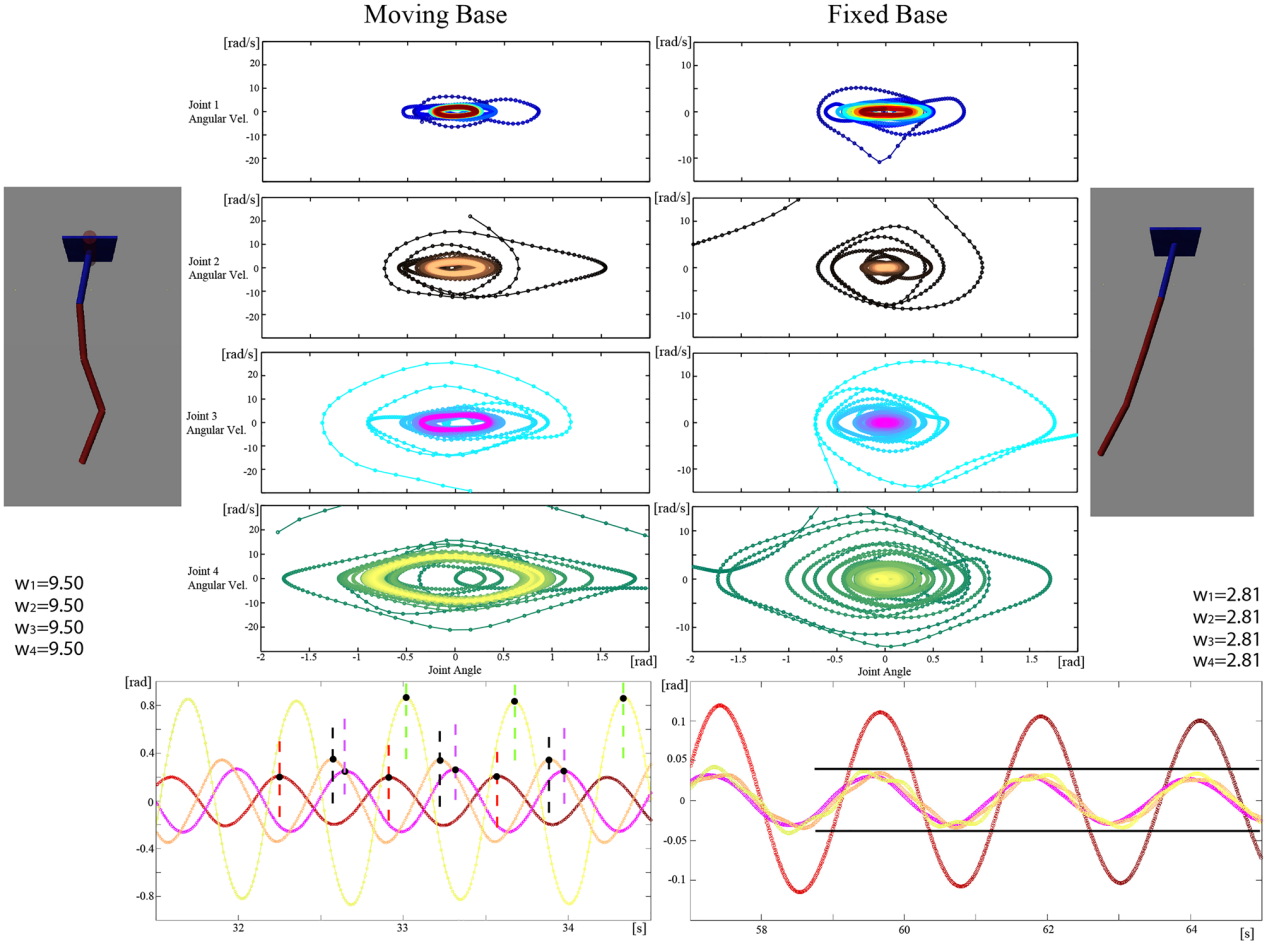
Joint angles of a four-segment pendulum in series. (**A**) The base link was a floating base. Coordinated motor patterns resulted in a synchronized frequency over all the joints. In the floating-base case, traveling wave coordination could be observed. (**B**) The base link was fixed. Here, in-phase coordination could be observed. The learning controller found synergetic coordination while adapting to the environment, without relying on neither the model information nor the coupled oscillator. Movie S4 shows the detailed process of how the limit cycle was formed. When the environmental situation allows, neural intergrator solution was converged into simple in-phase synergetic coordination with the same amplitude as in the fixed base result. The amplitude, frequency, and phase modulation then appear in the moving base to sustain the oscillation as the total system oscillation can not be managed when the oscillation amplitude is same over the segments.

To investigate a further complex situation and the feasibility of the adaptive capability to complex dynamics (Requirement 4), the proposed method was tested with a four-series segment pendulum. The first joint is passive and the three following joints were active. The pendulum was hung horizontally at the moving base. The system has highly nonlinear behavior and a chaotic relationship with the four segments interaction torques. Although the system started with chaotic oscillation, it converged to the limit cycle with synchronized frequency oscillation with the four joint angle variation having the same frequency as in Fig. 3. It converged to a reasonable phase delay (traveling wave) and with varied amplitudes (from small to large from the first joint to below). When the base link was a floating moving base, the base could be moved as a reaction effect if joint 1 applied the force, resulting in loss of energy. In contrast to the previous example, as there is only one pendulum at base, the interaction by using two pendulums moving in the opposite direction cannot be cancelled. Thus, the lower joints oscillate with higher amplitudes as in Fig. 3. The first upper joints have a smaller oscillation amplitude. To cancel the interaction force at joint 1, the phase delay was in anti-phase between joint 1 and joints 2 and 3. The situation allows joint 4 to move in-phase with joint 1 and to make large oscillation at the end. It is important to note that this adaptive behavior appeared from the simple distributed neural integrators, which does not use any state information from the other neighboring joints. For the case of a one serried-segment pendulum without moving the base, it is a reasonable choice to increase the oscillation amplitude for the bottom and decrease the oscillation amplitude for the upper joint. In the same system, a totally different joint coordination appeared with all joints in the in-phase state when the base link is fixed. This joint coordination is because it is not necessary to cancel the interaction force at the base link since there is no risk of energy loss when the base is fixed. The in-phase move over the joints is thus efficient in terms of energy as it did not cancel the accelerated segments. The joint move was then in-phase and the joints moved together in a synergetic manner with same amplitude of active joint. The proposed method was also effective for multi-joint articulation and synergetic synchronization; thus, this simple neural integrators can manage different complex conditions. With the same controller with a fixed or moving base, the adaptive joint coordination appeared for maintaining efficient synergetic oscillation. The oscillation frequency was also altered in a different base condition. The amplitude, phase, and frequency modulation appeared with a simple distributed integrators. Thus, adaptive and limit-cycle oscillation could be created, without any so-called coupled oscillator model.

## Discussion

Finding an adaptive oscillation controller which is energetically efficient in the given dynamics condition is not a trivial problem as the balanced energy should be injected by neural inputs to form dynamic equilibrium in energy dissipation system. When using conventional coupled oscillators, the amplitude, frequency, and phase parameters should be tuned; however, managing these parameters in an unknown environment is quite challenging. The selection of an oscillator model type is also a difficult issue. This study verified if simple distributed neural integration could be applicable to find limit-cycle inducing input patterns for unknown dynamical system. The results give us some inspiration on how neural integrators in living systems^27^ may manage the amplitude, frequency, and phase modulation corresponding to the given dynamics. In this report, in a different pendulum environment, the proposed method could manage to find the synergetic synchronization in various ways: (1) In-phase synergetic coordination in double pendulum as shown in Fig. 1. This provides insight on how to find arm or leg synergetic swing coordination. (2) Anti-phase coordination over two pendulums as shown in Fig. 2. This provides insight on how to manage two arms or legs swing phase modulation into anti-phase over limbs, and (3) The amplitude modulation with phase delay modulation over segments provides insight on how fish, snake or lamprey swim. By carefully observing the amplitude of the swim form of lamprey, the oscillation of the head can be seen to have a much smaller amplitude than the tail^24^. This adaptability and simplicity of the method should be appealing on the problem to find the synchronized oscillation that results in all joint oscillation convergence into the same frequency, even with the distributed control which does not have any frequency parameter. Regarding the condition on the self-organization for finding the control gain setting, it should be further studied in the future work. As same as newly proposed control methods, its limitation and the detailed analysis are expected to be performed for better understanding.

Like the gait of a biped or quadruped, if the basic relationship of the joints is known, the coupled oscillator method can show good performance. However, in order to answer how animals or insects can find its basic coupling relationship, the model-free approach can be beneficial to address the organization of CPGs without requiring knowledge of the coupled oscillator. The coupled oscillator is still important approach to be considered as one of the neural network of living system. However, before finding appropriate coupling relationship, we may need simple distributed neural integrators to find the coupled relationship of the given system. This method provides a new insight for the emergence of dynamic equilibrium in energy dissipation systems. This method can also contribute to the understanding of human/animal motor control for adapting to the environment with neural intergrator function.

When the model and the dynamic environment is unknown, finding the spatio-temporal control input pattern is not a trivial problem, as the cycle energy balance should be found in unknown environment. As a current engineering solution, reinforcement learning, which is reward based with huge amount of trial-errors, is a kind of standard approach. However, it requires too many data samples with trial-errors. If the dynamic equilibrium solution can be found with computationally non-expensive method, it would be quite useful to be combined with it. This paper results demonstrated even for complex dynamics problem with totally unknown environment, the dynamic equilibrium could be induced with simple distributed integrators. Thus, it would contribute to the motion controller design for reducing computation time to adapt to the unknown dynamical systems through bio-inspired neural integrators.

## Methods

Synergetic Learning control (SyLC) scheme for oscillation emergence can be represented with recurrent style neural integrator as below and it is extremely simple and with distributed manner. The first feedback term is simply making proportional control. For the Joint n, if the the joint is active, by using the current joint position, the distance to the center line is used as the error signal, the feedback signal is based on the error. The torque to rotate to cancel this displacement is generated by this feedback term. Importantly the feedback gain is set as small, then only feedback term can not sustain the oscillation. The 2nd term is the joint damping which is a source of energy loss during oscillations. The neural integration term in the synergetic learning control can form the feedforward control to adapt to the given dynamics. This term is formed to keep the total system energy for forming the limit cycle. However, differently from oscillator model approach, any phase relationship or coupling information between neuron or joint is not used in the proposed method (Requirement3). This framework needs only the self state information.

PD feedback case:

1 $$\begin{aligned} \tau _i=-k(\varvec{J_{i-1}} \varvec{\theta })_x-D\dot{\theta _i}. \end{aligned}$$

**Figure 4:**
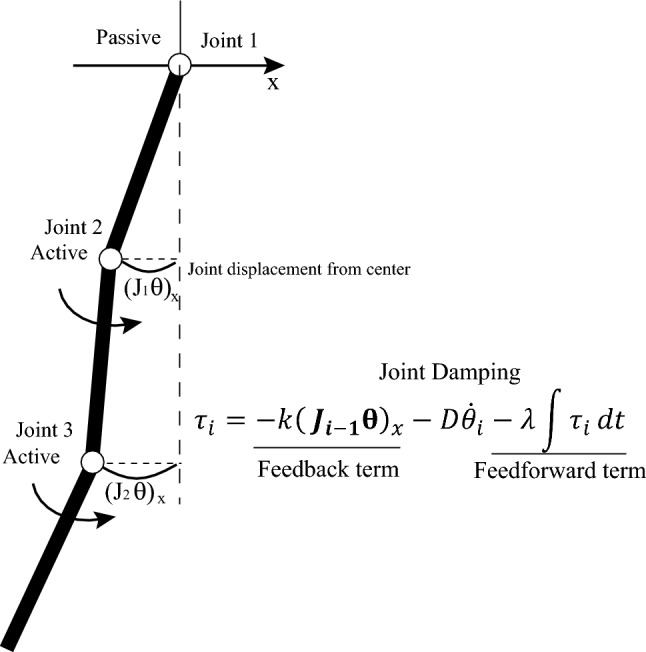
Synergetic learning control method for keeping limit-cycle oscillation.

SyLC case:

2 $$\begin{aligned} \tau _i=-k(\varvec{J_{i-1}} \varvec{\theta })_x-D\dot{\theta _i} - \lambda \int {\tau _i}dt. \end{aligned}$$

where $\tau _i$ and $\dot{\theta _i}$ denotes the control torque input and angular velocity of the i th joint. $\varvec{\theta }$ denotes the vector of the joint angle, $\varvec{J_{i-1}}$ is the Jacobian matrix up to the previous link. $(\varvec{J_{i-1}}\varvec{\theta })_x$ is simply the x axis distance from the center line as indicated in Fig. 4. This distance can be considered as error signal to be integrated over the time. *k* is the gain of the proportional feedback, *D* is the damping coefficient. $\lambda$ is a gain of the neural command integration, which is torque command in this study. The controller has no information on the dynamics parameter of the environment (Requirement1), and the control and learning are executed in a simultaneous manner (Requirement2). More details regarding the method are provided in the Supporting Online Material.

We assume only forward kinematics (FK) information is available. However, it is simply the joint position. Jacobian matrix up to the previous link multiplied with joint angle vectors up to the previous joint angle can compute the corresponding joint position. We need basically only this part of the information. This formula is common even for double pendulm or 4 series segment pendulum. For double pendulum, there is only 2 link, and for the 2nd joint position, it is decided only with 1st joint angle and the jacobian, which is just 2nd joint position itself. The active torque of the 2nd joint is controlled with the feedback of its position plus the negative integration, it could find the limit-cycle. Without feedforward, it can not find the limit-cycle. For the case with 4 series, for eg. 3rd active joint uses simply the feedback signal of the current position and the negative integration. Then, except the current position, the conroller at each joint doen’t use the other joint state information. Thus, it is totally the decentralized control.

Interestingly, we have used similar learning controller previously for multijoint reaching task^28,29^. At that time, the sign of the torque signal accumulation was positive. The solution was to have converged solution to follow the target by multi-joints system and to find energy-efficient solution by removing unnecessary energy usage. In this paper, for the oscillation emergence purpose, the opposite sign was employed for this feedforward term. It could be said that for promoting the target reaching, it was positively integrated toward the static equilibrium point at the target for reaching task. In contrast, the negative sign was well suited for making oscillation for dynamic equilibrium generation by keeping original system energy. The multijoint dynamics information is not given to the learning controller, thus this paradigm is to find a way to manage interaction torques through the repetitive interactions with the environment.

## Supplementary Information

Supplementary Movie S1.

Supplementary Movie S2.

Supplementary Movie S3.

Supplementary Movie S4.

Supplementary Information 5.

## Data Availability

The datasets used and/or analysed during the current study available from the corresponding author on reasonable request.
