# Supplementary material for: Synergetic synchronized oscillation by distributed neural integrators to induce dynamic equilibrium in energy dissipation systems
Source: Sci Rep. 2022 Oct 13;12:17163. doi: 10.1038/s41598-022-21261-w (PMC9563046; doi:10.1038/s41598-022-21261-w)
Supplement: Supplementary file 5 — Supplementary Information 5. [file 41598_2022_21261_MOESM5_ESM.pdf]

Supporting Online Material for

Synergetic synchronized oscillation by distributed neural integrators to induce dynamic equilibrium in energy dissipation systems

Mitsuhiro Hayashibe*,

Shingo Shimoda

*To whom correspondence should be addressed. E-mail: hayashibe@tohoku.ac.jp

This PDF file includes:

Supplementary information regarding Materials and Methods, Figs. S1, S2, S3, S4 and Captions for Movies S1, S2, S3, S4.

Materials and Methods

1.1 Synergetic learning control with neural integrator

Synergetic learning control scheme for oscillation emergence can be represented as in Fig. S1. The First feedback term is simply making proportional control. For the Joint n, if the the joint is active, by using the current joint position, the distance to the center line is used as the error signal, the feedback signal is based on the error. If the joint position is on the left side as in Fig. S1, the torque to rotate to cancel this displacement is generated by this term. However, importantly the feedback gain is set as small, then only feedback term can not sustain the oscillation. To verify this setting, we make simulation always only with the feedback term to confirm the oscillation is to be stopped only with this term before adding the learning feedfoward term.

The gain is constant, then essentially this feedback term can not regulate the energy injection to have dynamic equilirium. If the small gain, it makes small injection of energy, and we have certain joint damping, then the oscillation is being stopped as shown in the result and the video at the supplementary materials. If the large gain, it may make large injection but in this case, the energy diverges and it is not possible to generate limit-cycle oscillation.

The second term is the joint damping embeddded in all the joint including passive joint at the root. The third term is the neural integrator for torque signal accumulation. The multijoint dynamics information is not given to the learning controller, thus this paradigm is to find a way to manage interaction torques through the repetitive interactions with the environment.

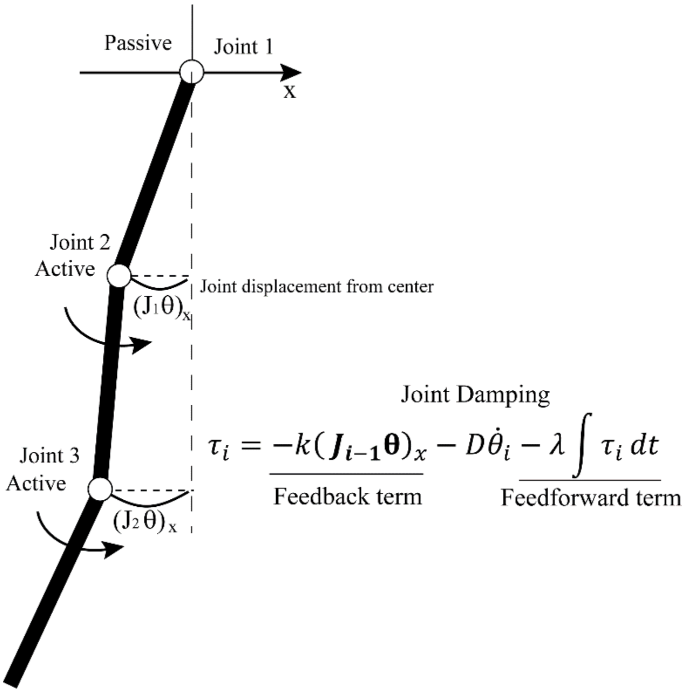

Fig. S1. Synergetic learning control method for keeping limit-cycle oscillation.

The motor pattern integration term can be considered as FF controller which anticipates the environmental interactions during oscillation. We should note that even when the integration term is represented as I in the diagram, this control structure is totally different from so-called PID structure where the joint reference error is normally integrated. Instead, in the proposed method, the mapped motor field comprising each joint motor pattern is being integrated in a modular configuration. This integrated motor pattern has cyclic torque signature then this term can still continue to send predictive motor command independent from the feedback information. This is the reason why this term can be regarded as FF controller. During the learning, the contribution from FF was increased and the torque from FF was converged into certain pattern.

1.2 Experiment on real system

In the sagittal plane, three degrees of freedom (DOF), composed of the shoulder, elbow, and wrist joints, were arranged as illustrated in Fig. S2(A). The upper arm, forearm, and hand segments were connected through each joint. Each joint is actuated using a DC motor with an encoder and a harmonic drive gearing. With Joint 3 fixed and Joint 2 only actuated, Joint 1 was made to have a compliant mode, which emulated the passive joint. 10 W motors were used for Joint 2 (elbow). The ratio of the gears were all 1/100. The motor was current-controlled with servo-amplifier drives. Thus, each joint had a local torque control to generate the specified joint torque by the synergetic learning controller for the robot. The control algorithms were executed with the sampling frequency of 500 Hz on a master PC with the interface of AD and DA converters from the encoders and to the motors, respectively. As in Fig. S2(B), the end-point weighed 450 g to give mass to the robot. The experiment was to check the performance equivalent to one double pendulum simulation as in Fig. 1. Therefore, joint 1 was passively moved and the active torque was applied to joint 2 for comparison to the first simulation in the main body.

Movie S4 shows this experimental result. The first sequence shows that without learning only with the feedback term, the oscillation cannot be sustained by the damping effect. The plot of Fig. S3 corresponds to this video segment. The second sequence with learning found the limit-cycle, and some disturbances by hand, was applied to the system. However, after some time, the system recovers the same limit cycle. Fig. S4 corresponds to this video segment. The angular velocity and the convergence to the same limit cycle with the same frequency as the learning controller are confirmed. Even with the provision of a complex interaction by hand to the real system, it recovered into the same rhythm. This result shows the feasibility of the method to the real system. Although different from the simulation, the real joint friction and damping may not be linear and unknown. However, the method could be used to produce a limit cycle that requires a dynamic equilibrium mechanism.

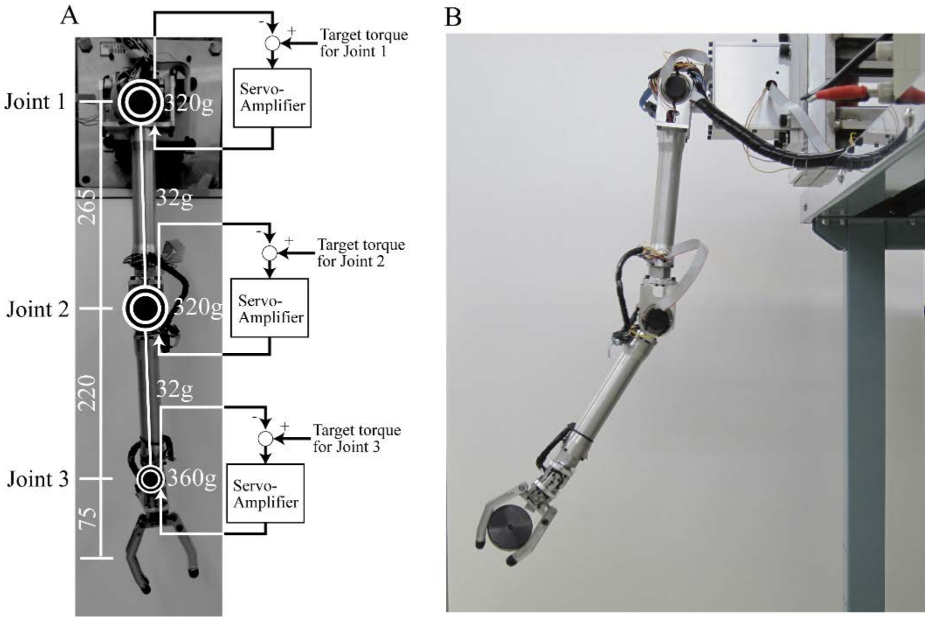

Fig. S2. Manipulator configuration used for experiment. Joint3 was fixed, Joint2 was only actuated, and Joint1 was made to have a compliant mode, which emulated a passive joint.

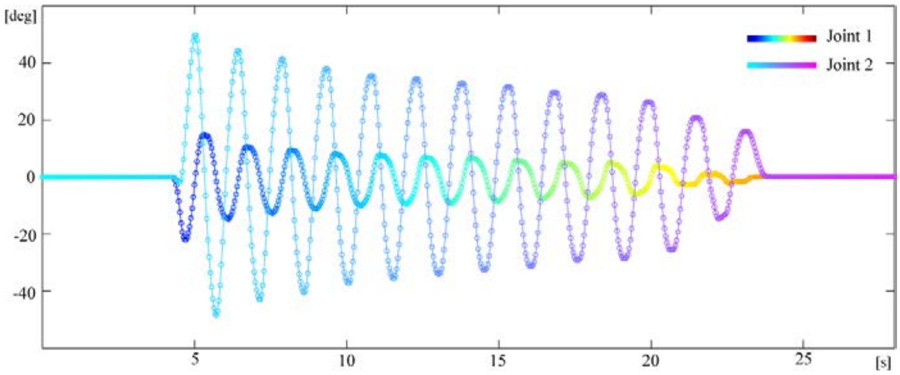

Fig. S3. Joint angle changes only with feedback term and without learning term. This figure corresponds to the results of Movie S4.

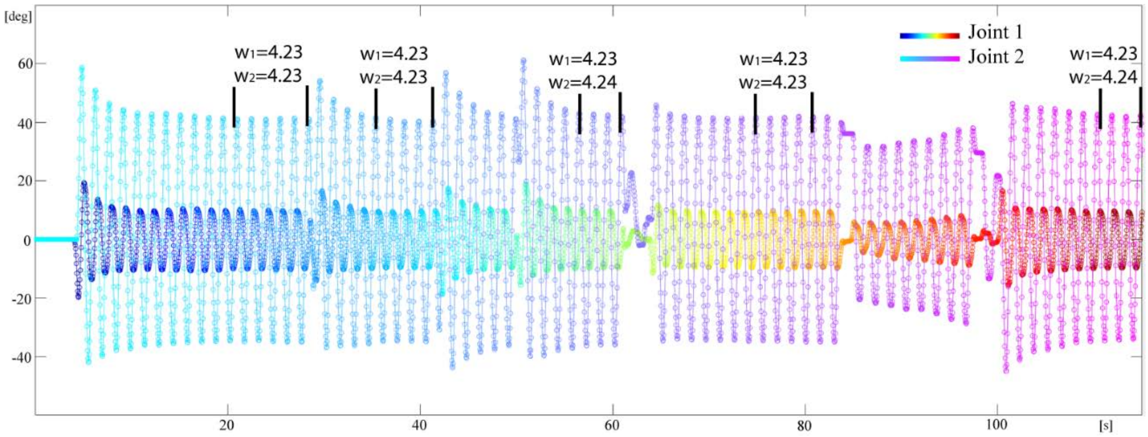

Fig. S4. Joint angle changes with learning term, corresponding to the results of Movie S4. As disturbance was added, it turns to the limit cycle. By checking the steady-state part, the two-joint motion frequency was synchronized with the in-phase motion.

References

Russell Smith (2000). Open dynamics engine. Available online at: http://www.ode.org/

Movie S1.

This movie depicts the animated simulation results for the double pendulum, as shown in Fig. 1. The first sequence shows that when learning is not used to show the energy dissipation by damping, the oscillation cannot be sustained; this is followed by the case of learning producing the limit cycle. The longer limb case converges to a slower motion frequency.

Movie S2.

This movie depicts the animated simulation results for two double pendulums with a common base, as shown in Fig. 2. When the base is fixed, it similar to two independent double pendulums as in Movie S1. There is no phase modulation over two sets of pendulums. When the base is moving, the anti-phase motion appears, which minimizes base movement, even if the base is not fixed. This supports energy preservation.

Movie S3.

This movie depicts the animated simulation results for a four-segment pendulum in series. The first sequence shows the case with a moving base. In this case, a different amplitude creates a smaller displacement on top to minimize base movement and phase delays like a traveling wave. The second sequence shows the case with fixed base. The behavior changes to in-phase and same amplitude for active joints, which is more energy-efficient for maintaining oscillation when the base is fixed. The base is not a source of energy loss.

Movie S4.

This movie depicts the results of a robot experiment of one double pendulum to verify the feasibility of this method to real-world applications. There is unknown joint friction and damping. The first sequence shows that without learning and only using the feedback term, the oscillation by the damping effect cannot be sustained. The second sequence with learning found the limit cycle. Some disturbances were applied manually to the system, but after some time, the same limit cycle was recovered.
